# Only one health, and so many omics

**DOI:** 10.1186/s12935-015-0212-2

**Published:** 2015-06-23

**Authors:** Nives Pećina-Šlaus, Marko Pećina

**Affiliations:** Laboratory of Neuro-oncology, Croatian Institute for Brain Research, School of Medicine University of Zagreb, Salata 12, HR-10000 Zagreb, Croatia; Department of Biology, School of Medicine, University of Zagreb, Salata 3, Zagreb, Croatia; Department of Medical Sciences Croatian Academy of Sciences and Arts, Zrinski trg 11, Zagreb, Croatia

**Keywords:** Omics, Genomics, Proteomics, Epigenomics, Metabolomics, Microbiomics, Cancer

## Abstract

The development of new approaches based on wide profiling methods in studying biological and medical systems is bringing large amounts of data on a daily basis.

The causes of complex diseases have been directed to the genome examination bringing formidable knowledge. We can study genome, but also proteome, exome, transcriptome, epigenome, metabolome, and newcomers too such as microbiome, connectome and exposome. The title of this editorial is paraphrasing the famous saying of Victor Schlichter from Buenos Aires children hospital in Argentina who said “How unfair! Only one health, and so many diseases”. Today there is indeed a whole lot of omics. We think that we are lucky to have all the omics possible, but we also wanted to stress the importance of future holistic approach in integrating the knowledge omics has rewarded us.

## Introduction

The development of new approaches based on wide profiling methods in studying biological and medical systems is bringing large amounts of data on a daily basis.

The causes of complex diseases have been directed to the genome examination bringing formidable knowledge. Cancer is a very complex disease. For a long time now we know that genetics is standing behind cancer etiology and genome examination is still primary approach in studying all types of cancer. Nevertheless, other cellular and biochemical levels are equally important. We can study genome, but also proteome, exome, transcriptome, epigenome, metabolome, and newcomers too such as microbiome, connectome and exposome. The high-throughput techniques that we nowadays employ have changed cancer research [1]. They allow wide illustrations of different levels of the specific problem.

## Review

The most common *omics* employed in molecular cancer research are genomics -the analysis of genome structure of organisms as a whole [2, 3]. Genome can be defined as a complete genetic material of an organism the complete nucleotide sequence of its DNA. The human genome is comprised of 3.2 billion nucleotides, but contains only 23,500 protein-coding genes [4, 5]. Closely connected to genomics are exomics and transcriptomics. Exomes are the protein coding content of the genetic code, the part of the genome formed by exons. The human exome consists of 180,000 exons, roughly 30 Mb of DNA which is around 1-2 % of the total genome [6, 7]. In comparison to whole-genome sequencing meaning decoding 3.2 billion nucleotides, exome sequencing is much quicker, cheaper and far more comprehendable [8, 9]. Approximately 99 % of the human genome does not code for a protein. Nevertheless, we know now that all this amount of sequences are also important in performing different functions - some known and some unknown [5]. The difference between exomics and transcriptomics is basically the fact that transcriptome encompases all RNA molecules synthesized by the process of transcription, while, as we pointed out earlier, the genomes and exomes are at the DNA sequence level with relatively fixed nature [10]. The non-fixed nature of transcriptome is reflected in different rates of transcription *e.i.* synthesis of RNA molecules in a specific organism, tissue or cell type at a given time. Besides mere presence of a given RNA molecule, transcriptome also informs us on its amount at certain time and place. Therefore it shows the expression of the information encoded by the genome. The regulation of expression levels are influenced by different intrinsic signals and stimuli but also as a response to environmental conditions enabling cell to respond and adapt. Traditionally transcriptome is analized by cDNA microarrays, but in the last several years novel next-generation sequencing platforms also known as RNA-seq techniques [11, 12] are used.

The high through-put omics data in cancer are providing us with information often referred to in a number of scientific papers as genomic landscapes of cancer [13, 14]. This excellent term encompasses multitude of specific genetic events and aims to illustrate the whole complex cancer system [15]. Since we are talking about landscapes there should be an atlas. Therefore, the Cancer Genome Atlas (TCGA) project began in 2006. by collaboration of National Cancer Institute and National Human Genome Research Institute. This endevour aims to assemble all known changes for about 20 cancer types [16]. In order to enable researchers to search, analyze and validate important discoveries the data are publicly accessible through the Cancer Genome Atlas (TCGA) Data Portal. It is noteworthy to mention another important project searching for somatic alterations in cancer. The Cancer Genome Project of the Wellcome Trust Sanger Institute. This ongoing project is using the human genome sequence and high-throughput mutation detection techniques to identify somatically acquired DNA sequence mutations in human tumours and tumour derived cell lines. Through its resources, The Cancer Gene Census and Catalogue of Somatic Mutations in Cancer (COSMIC), the project aims to systematically catalogue genes mutated in human cancers thus identifying genes responsible for cancer development [17–19].

The resulting integration of omics data showed both subtype-specific genetic profiles but also similarities and common changes shared among different cancer types. We can now distinguish driver from passenger mutations, a concept which can explain the great heterogeneity of certain cancer types. The definition of a driver mutation is the one that confers a selective growth advantage to the tumor cell. Finding the driver mutations from passenger is the major challenge in cancer genomics. As an example, TCGA’s breast cancer project identified 30626 somatic mutations by whole exome sequencing of 510 tumors [16]. The somatic mutations included 28319 point mutations, and 2302 insertions/deletions. With such large number of aberrations it is hard to distinguish which are key driver mutations and which signaling pathways play major roles. Therefore recurrent mutations more frequently found across specific tumors are proclaimed as driver mutations. This is easily determined when the number of mutations in a gene and the frequency of affected gene is very high, as with *TP53* or *KRAS*. Nevertheless, mutation frequency is not entirely reliable approach in driver genes identification. There are many genes with more than one, but still relatively few mutations. In these cases, methods based on mutation frequency cannot reliably indicate which genes are drivers. Vogelstein et al. [14] state that the best way to identify driver genes is through their pattern of mutations rather than through their mutation frequencies. To classify a gene as a driver of oncogenesis it is important to characterize the gene as an oncogene or a tumor suppressor gene. The pattern observed from many functional studies shows that oncogenes are recurrently mutated at the same amino acid positions, while tumor suppressor genes are characterized by protein truncating mutations. So, the pattern of mutations characterizing an oncogene, is that >20 % of the recorded mutations are at recurrent positions and are missense, and to be classified as a tumor suppressor gene, >20 % of the recorded mutations in the gene are inactivating.

Passenger mutations encompasse all those neutral mutations that have been accumulated in the original cell before the oncogenic event occured [1]. They also happen along the way of clonal expansion and tumor progression but are not directly or indirectly involved in the selective growth advantage of the cell in which they occurred. They can occur during the preneoplastic phase having no effect on the neoplastic process. Nevertheless, they are very important for metastatic processes, for patients response to therapeutics and the clinical course of the disease.

There are several computational models and algorithms employed for functional consequence prediction of detected mutations [15]. There are also computational models and algorithms for data integration across cancer-types and for data provided by different platforms. The statistical assumptions used have been the subject of debate, since it is difficult to determine a background mutation rate for each tumor type. The development and improvement of adequate computational methods for interpreting genome-scale molecular information are urgently needed today since the true meaning of complicated data still needs to be elucidated.

To sum up what we have learned from genomic data on cancer we can say that we know that in common solid tumors an average of 33 to 66 genes display somatic mutations affecting their protein products. However, there are tumor types that display many more mutations, but also those that have only few mutated genes. COSMIC’s latest release (v71; Sept 2014) describes 2 710 449 coding point mutations in over one million tumor samples and across most human genes (28 977). About 95 % of these mutations are single-base substitutions of which more than 90 % are missense mutations, whereas the remaining are deletions or insertions of one or a few bases. It is estimated that there are only about 138 mutation driver genes across the cancer landscapes. Of these, 74 are tumor suppressor genes and 64 are oncogenes [14]. The complexity of cancer genomes is overwhelming, displaying great genetic heterogeneity even within the same histopathological tumor type. A palette of driver mutations together with passenger mutations make each individual tumor distinct. So how can we make sense of such a great variability? The answer and the light at the end of the tunnel lies in the fact that the signal transduction pathways affected in different tumors are similar. So, when the affected genes are placed into signaling pathways, the cancer complexity is significantly reduced [13]. The specific or shared pathways in significant numbers of tumors can now be outlined. All of the known driver genes function through 12 core signaling pathways: WNT, NOTCH, Hedgehog, TGF-beta, MAPK, STAT, PI3K, RAS, chromatin modification, transcriptional regulation, DNA damage control and cell cycle-apoptosis [14, 20].

The observed pathway similarities will provide opportunities to design tumor treatment targets and therapeutic discoveries. It seems that it is not crucial to disable driver genes, but is sufficient to interfere with the affected pathway in order to treat cancer. Therefore we can now start designing treatments according to the implicated pathway and not only to inhibit the action of many specific individual proteins encoded by mutated genes. Deep functional validation of candidate cancer genes is still necessary before genomics information can be introduced into clinical practice. It is important to understand that not all somatic mutations within the gene will be functionally equivalent. Clinical application of genomics will soon refine the current cancer diagnostics and classification providing more accurate biomarkers and personalized treatments. Diagnostics will improve by development of new sets of specific tests for each tumor type based on mutated genes and affected signaling pathways.

In the past decade formidable data were brought by proteomics, a field which can be defined as a large-scale study of proteins, their functions and structures [21]. Proteomics alone is a system science. Since proteins are functional building blocks of cells, the information on proteome of a given cell or tissue in health or disease, through different phases of cell’s life, in interaction with the environment, *etc.* is a difficult but rewarding task to accomplish [22, 23]. The Human Proteome Project (HPP) aims to map the entire human proteome and the instrumentation employed to do this is mass spectrometry and bioinformatics [24, 25]. Human proteome consists of 23,500 protein coding genes, but we have to add different protein isoforms that are estimated to million different protein isoforms, meaning million different proteins [26].

Proteome profiles characteristic of specific cancer pathology will open new horizons in cancer research. Changed cancer genome is reflected at the functional level which means that proteome of a cancer cell is changed. Comparation of cancer proteomes can be approached at two directions, absolute quantitation of observed changes and relative approach where comparison of relative changes are measured. Absolute amounts of proteins in a certain sample are much harder to define and obtain, therefore relative changes are usually measured in most proteomic experiments.

One must not forget about epigenomics, the field that counts all epigenetic changes, the changes, as the word says, that are above the genome. The changes that are above the nucleotide sequence of our genome include molecular mechanisms of the modification of DNA and the modulation of chromatin structure. Those mechanisms can modify gene expression in differentiated cells [27]. Methylation of cystein residues at specific positions in the DNA is the premier molecular mechanism associated to epigenetic changes. Maintenance of methylation patterns is important regulatory element and is closely connected to genomic imprinting, a phenomenon where expression of certain genes depends on whether they are maternally or paternally inherited. Besides methylation, epigenetic mechanisms also include post-translational modifications of histones, chromatin remodeling and non-protein coding RNA interfering pathways [28, 29]. The study of epigenome aims to decipher and comprehend these collaborative epigenetic modifications in order to understand transcriptional regulation and establish precise gene expression programs.

Human tumors contain large numbers of epigenetic changes affecting DNA or chromatin proteins. That epigenetic events may be involved in carcinogenesis is reflected throught a large number of genes that are aberrantly expressed without being mutated. As the tumor evolves those genes display changes in DNA methylation or chromatin modification. Moreover, it has been shown that a great number of driver genes encode proteins that regulate chromatin remodeling.

The first discovery of epigenetic alterations in cancer happened some 30 years ago when global DNA hypomethylation was reported in cancer in comparison to normal cell [30, 31]. Global hypomethylation in cancer is referred to a decrease in overall content of 5-methylcytosine found in cancerous tissues. Hypomethylation usually targets repetitive sequences and is observed not only in cancer but also in non-cancerous tissues exposed to chronic inflammation. However, exactly how epigenetic alterations are induced by exposure to inflammation is still not understood. Later on promotor hypermethylation was discovered to cause silencing of tumor suppressor genes. Recent genomic research discovered mutations of epigenetic regulators in cancer [31]. For example, frequent mutations of IDH1 and IDH2 regulators were found in gliomas which lead to loss of their function – genome wide alterations of histone modifications. Mutations of other epigenetic modifiers have also been identified usually resulting in aberrant histone methylation [31].

Is the altered expression of epigenetic gene a driver or a passanger event and are specific epigenetic changes causes or consequences of tumorigenesis? Those are questions that still need to be answered.

The difference between genetic and epigenetic changes is that the genetic sequence is fixed while methylation is plastic and dependable on microenvironment, patient age, nutrient concentration *etc.* [14, 32]. Aberrant epigenetic events are frequently observed in early-stage cancers and in adenomas. Thus, epigenetic epidemiology has great application in cancer prevention by identifying risk factors and establishing markers of early disease [33]. One of the most important characteristics of epigenetic alterations is that they can be reversed. This characteristic can serve us well in the development of epigenetic drugs for the purpose of restoration of normal epigenome. Candidates for epigenetic drugs are DNA demethylating agents, inhibitors for histone methyltrasferases and histone demethylases and proteins that recognize histone modifications.

The newcomers in omics are very interesting too - metabolomics - the study of the complete set of all metabolites in an organism, microbiomics - the study of the microbes in our gut and body and how they might cause certain conditions, connectomics- the study of connectomes with ultimate goal to map all the neural connections of human brain, exposomics, defined as the totality of exposures received by an individual during a lifetime.

A metabolome is defined as the complete set of all metabolites in an organism. Metabolites are low molecular weight molecules less than 2000 Da in size and are the intermediates and end-products of metabolism. Within this context the metabolomics is the study of the complexity and totality of small molecular metabolitic intermediates [34, 35]. The metabolome in contrast to genome and proteome is very dynamic and susceptible to quick changes since it is influenced by environment, microbiota and other different physiological stimuli. Therefore, in contrast to genome and proteome, metabolome is difficult to define. The main analytical techniques employed in metabolomics are nuclear magnetic resonance spectroscopy (NMR) and mass spectrometry (MS) usually measured from biofluids such as plasma and urine [36]. In 2007. human metabolome was described and the corresponding Human Metabolome Database was established [37]. Human metabolome consists of a great number of endogenous and exogenous compounds. Endogenous being synthesized by the enzymes encodes by our genome and exogenous represent foreign chemicals consumed by our body that all have effects on metabolic fluxes and metabolic pathways.

When talking about metabolomics we have to consider it in relation to metabolic control theory also know as flow theory formulated about 40 years ago by Kacser and Burns [38]. The theory describes how metabolic fluxes and concentrations depend on enzyme amount and gene dose. It is based on measuring changes in steady-state metabolite concentrations and fluxes induced by parameter modulation [39, 40]. The authors promoted the operational definition called the flux control coeffieient, the relative increase in flux, divided by the relative increase in enzyme activity that brought it about. The theory provided great improvement in our understanding of the control of metabolism. Actually, the hyperbolic-like relationship between enzyme activity and flux seems to be valid for most of the networks including complex networks, regardless their complexity [41]. Fluxes through metabolic networks can be considered as model quantitative trait, depending on all the genes coding and regulating the enzymes of the network [40].

Concentration is a key parameter for enzyme activity and changes in expression of enzyme genes play a central role in the physiology of the cell. It has been used to describe the response of metabolic concentrations and fluxes to infinitesimal changes in enzyme concentrations and effectors [42].

All pathways are inter-related, some closely and others more distantly, i.e. everything in a cell is connected. But the closeness of the relationship can change as the cellular environment changes. This means that intermingling pathways might come in and go out depending on the conditions - which gene starts operating differently affecting other gene expression. So, any enzyme in a biochemical pathway can become rate limiting, thus controlling metabolism [43, 44].

The metabolic theory also provides explanation for why so many large-effect mutations are recessive. Kascer and Burns hypothesized on methematical grounds and on the basis of empirical data that the relationship between flux through a long metabolic pathway and enzyme activity at any sigle step in the pathway is a curve of diminishing returns. If we view the organism as an enzyme system consisting of a large array of catalysts organized into diverging and converging pathways and resulting in a plow of metabolites as Kascer and Burns stated themselves [45] the recessivity of large-effect mutations can be explained as a consequence of a diminishing relationship betweeen flux through a metabolic pathway and enzymatic activity at any step in the pathway [46]. For several years after the flux theory was proposed, this method was little used, but later on it was extended by a number of groups and applied to various systems [47]. Perhaps it should be rediscovered in omics millieu, too.

Closely connected and influencing metabolomics is the new field of microbiomics. Microbiome represents all genomes of microorganisms (or microbiota) that symbiontically live in us or on us. The magnitude of human microbiota is overwhelming. It consists of about 100 trillion microbial cells ten times outnumbering human cells [48, 49]. Additionally the number of genes in the microbiome may exceed the total number of human genes by two orders of magnitude. Our microbial symbionts therefore have high influence on our biology. For instance microbiomics are actively involved in the control of host metabolism and immune system development. Human intestinal microbiota can be regarded as a new organ capable of performing numberous biochemical processes. Different microbiota inhabit different body sites and the knowledge of composition of microbial communities at specific site is important in order to recognize changes due to diseases [50]. The typical approach in microbiomics study is to choose a marker gene present in all of the investigated micororganisms and yet whose sequences are variable enough to be able to distinguish taxonomies. The marker gene of choice is small subunit ribosomal RNA (16S rRNA) gene [51]. Enabled by low-cost, high-throughput DNA sequencing and on the basis of 16S rRNA sequences microbiomics is analyzing and collecting thousands of microbial DNA sequences.

Another omics that is not directly related to cancer research but nevertheless needs to be mentioned is connectomics. Because it will definitely have implications in central nervous system tumorigenesis and consequences of it.

Discovery of structural and functional brain connectivity at different spatiotemporal scales is brought by connectomics. How the brain really functions as a whole is still an enigma that connectomics aim to elucidate. From the network(s) of billions of neurons and synapses, all the way up to structural networks of cortical and subcortical regions at brain’s macro scale everything is connected by exchanging signals and influencing each other. The explanation of this dynamic network of interactions will yield vital data on neural pathways that underlie brain function, behavior and also individual differences [52].

The Human Connectome Project aims to map all neural connections within healthy individuals’ brains using neuroimaging methods (structural MRI, Resting-state functional MRI (rfMRI), diffusion imaging (dMRI) and Task-evoked fMRI). There are challenges connectomics face, for example this integrative map cannot capture modulatory processes and there are also questions on individual and temporal variability.

Another equally interesting newcomer is the exposome, the omics that aims to encompass total environmental exposures through person’s lifecourse from the conception onwards. Exposome started as part of epidemiological research where it was first introduced because of the need to assemble environmental exposure data. Closely complementing the genome, exposome is a compilation of non-genetic exposures influencing human health.

The multitude of environmental exposures varies from external sources which include radiation, chemical contaminants and pollutants in air and water, lifestyle factors, diet, occupation and medical interventions, noice, vibrations and climate. Nevertheless, endogenous processes like inflammation, oxidative stress, gut microbiota, diseases and infections are equally important [53–55]. When adding wider social economic factors and mental stress the challenge of exposomics’ impact on human health is even greater [56]. The study of exposome is performed by simultaneous measurment of a multitude of biomarkers from both sources [57]. The standardization of measurements is still a challenge. Nevertheless, the data brought by exposomics will contribute to better understanding of etiologies of human diseases and prevention. In todays view of disease etiologies it has been estimated that the majority of chronic diseases are attributed to environmental factors meaning that they are caused by exposome. One of the first attempts to measure early life exposome is the setting up of HELIX project [58]. The project aims to measure and integrate a wide range of chemical and physical exposures during pregnancy and infancy.

However, omics data may be highly variable and the results can easily be misinterpreted. Even genomics that has long been viewed as static have been shown to be plastic and liable to the dinamical changes under the influence from for example the environment. Another angle that can explain variabilty are different sample conditions, experiment preparations, instrumentation that all may influence the variabilty of omics results.

**Fig. 1:**
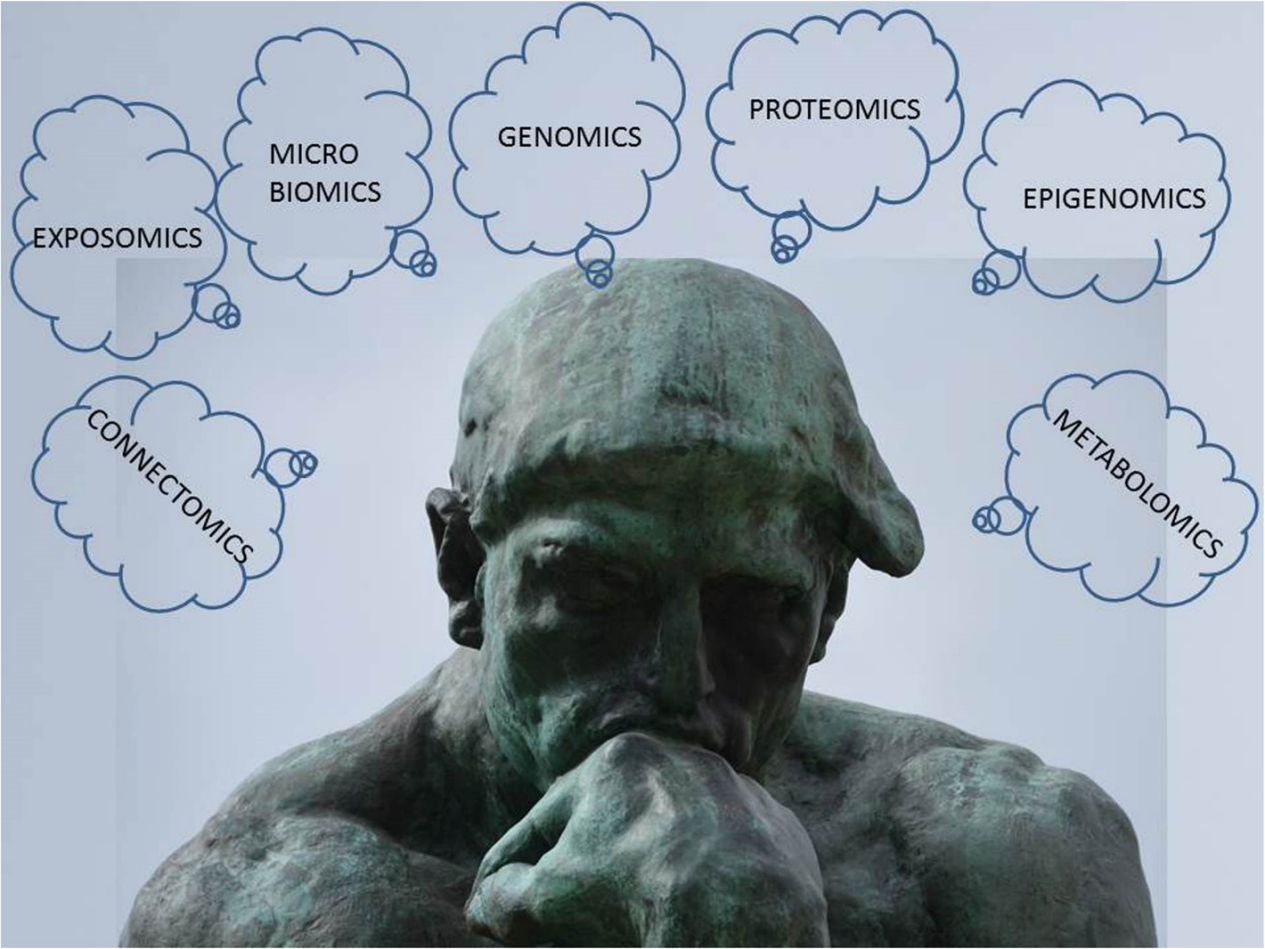
The thinker by Auguste Rodin. Vast amount of knowledge needs integration

The title of this editorial is paraphrasing the famous saying of Victor Schlichter from Buenos Aires children hospital in Argentina who said “How unfair! Only one health, and so many diseases”. Today there is indeed a whole lot of omics. I think that we are lucky to have all the omics possible, but I also wanted to stress the importance of future holistic approach in integrating the knowledge omics has rewarded us (Fig. 1). In molecular biology and molecular medicine one tends to compartmentalize the knowledge obtained at different levels. In our opinion this is not the deliberate action, but rather the consequence of very elaborate and sophisticated methods of data and information obtaining and also because of the fact that the field is still young. A lot of work is still ahead for the scientific community in analyzing and interpreting the data we have collected and in application of omics in clinical environment and diagnosis. Especially promising is the understanding of the development of cancer as well as the heterogeneity of this disease.

## Conclusion

Different sophisticated, detailed, compex and elaborate fields conditioned us to be experts only in single compartment of biology or even only in the specific level of it. As science progressed through centuries it became clear that one person cannont truly comprehend vastly different fields and we concluded that in science there are no more renaissance men. But in the light of new vast research and high-throughput techniques we will have to go back to the future and once again become renaissance men understanding biology, biochemistry, bioinformatics and biophysics. Only this time it is going to be much more difficult. We have data but now we have to make sense of it. Seems to us even harder to achieve.
